# Natural Shifts in Endosymbionts' Occurrence and Relative Frequency in Their Ciliate Host Population

**DOI:** 10.3389/fmicb.2021.791615

**Published:** 2022-01-11

**Authors:** Felicitas E. Flemming, Katrin Grosser, Martina Schrallhammer

**Affiliations:** Microbiology, Institute of Biology II, Albert Ludwig University of Freiburg, Freiburg, Germany

**Keywords:** killer trait, *Candidatus* Megaira polyxenophila, *Paramecium*, *Caedimonas varicaedens*, competition, occurrence, lake ecosystem, fitness

## Abstract

The role of bacterial endosymbionts harbored by heterotrophic *Paramecium* species is complex. Obligate intracellular bacteria supposedly always inflict costs as the host is the only possible provider of resources. However, several experimental studies have shown that paramecia carrying bacterial endosymbionts can benefit from their infection. Here, we address the question which endosymbionts occur in natural paramecia populations isolated from a small lake over a period of 5 years and which factors might explain observed shifts and persistence in the symbionts occurrence. One hundred and nineteen monoclonal strains were investigated and approximately two-third harbored intracellular bacteria. The majority of infected paramecia carried the obligate endosymbiotic “*Candidatus* Megaira polyxenophila”, followed by *Caedimonas varicaedens*, and *Holospora undulata*. The latter was only detected in a single strain. While “*Ca*. M. polyxenophila” was observed in seven out of 13 samplings, *C. varicaedens* presence was limited to a single sampling occasion. After the appearance of *C. varicaedens*, “*Ca*. M. polyxenophila” prevalence dramatically dropped with some delay but recovered to original levels at the end of our study. Potential mechanisms explaining these observations include differences in infectivity, host range, and impact on host fitness as well as host competitive capacities. Growth experiments revealed fitness advantages for infected paramecia harboring “*Ca*. M. polyxenophila” as well as *C. varicaedens*. Furthermore, we showed that cells carrying *C. varicaedens* gain a competitive advantage from the symbiosis-derived killer trait. Other characteristics like infectivity and overlapping host range were taken into consideration, but the observed temporal persistence of “*Ca*. M. polyxenophila” is most likely explained by the positive effect this symbiont provides to its host.

## 1. Introduction

Symbiotic interactions are important biotic factors which influence the realized ecological niche of organisms hence their occurrence and relative abundance (Chase and Leibold, 2003). Such symbiotic interactions can either be facultative or obligate and comprise the complete range from mutualistic to parasitic and from horizontal to mixed and vertical transmission (e.g., Fujishima 2009a), thereby determining the fitness of each partner, host as well as symbiont. Examples from the unicellular eukaryote *Paramecium*, a common motile ciliate living in the littoral zone of freshwater bodies, comprise endosymbiotic green algae providing photosynthesis products to their hosts and highly infectious intranuclear bacteria (Fujishima, 2009a; Schrallhammer and Potekhin, 2020). Efforts to unravel the diversity of *Paramecium*'s symbionts have resulted in the descriptions of several new bacterial species, genera and even new families and orders (Szokoli et al., 2016; Schrallhammer et al., 2018; Castelli et al., 2019; Beliavskaia et al., 2020; Korotaev et al., 2020; Castelli et al., 2021). Besides green algae (Flemming et al., 2020; Spanner et al., 2020), also microsporidians (Yakovleva et al., 2020), and fungi (Görtz, 1982) can occasionally be found as endosymbionts of *Paramecium*. For the majority of these associations, their effects are unknown.

Consequences and innovations of symbioses between *Paramecium* and its symbionts are versatile and include the acquisition of a mixotrophic lifestyle. An intensively studied example is the symbiotic interaction between *Paramecium bursaria* and its *Chlorella*-like algae (e.g., Fujishima, 2009a). The algal symbionts provide photosynthesis products like carbohydrates and oxygen as well as lipids, whereas the ciliate supplies Mg^2+^, CO_2_, and glutamine (He et al., 2019). The role of bacterial symbionts for heterotrophic *Paramecium* species is more ambiguous. Many if not most symbionts are obligate intracellular thus completely dependent on a host cell for reproduction. As they require resources from the host cell, these bacteria inflict costs on *Paramecium* (Dusi et al., 2014; Weiler et al., 2020) like reduction in cell division, motility, survival (Restif and Kaltz, 2006; Nørgaard et al., 2021; Zilio et al., 2020) as well as the potential disruption of sexual processes (Görtz and Fujishima, 1983). On the other hand, several experimental studies show that paramecia carrying a bacterial endosymbiont have an advantage relative to aposymbiotic or symbiont-free cells (Hori and Fujishima, 2003; Bella et al., 2016; Grosser et al., 2018; Schu and Schrallhammer, 2018; Koehler et al., 2019; Pasqualetti et al., 2020). In the following, the term aposymbiotic is used to describe cells which have lost their endosymbionts, e.g., by antibiotic treatment, hence, these aposymbiotic cells once harbored a bacterial infection which was subsequently eliminated. Cells collected from nature not harboring symbionts are referred to as symbiont-free.

In the past years, experimental testing of the effects of different abiotic stressors on ciliate-bacteria symbiotic associations has lead to a more detailed knowledge about specific aspects of those interactions. Symbiotic interactions can remain stable even under antibiotic pressure (Mironov and Sabaneyeva, 2020). Furthermore, interactions within a given symbiotic system can shift from mutualistic to parasitic and vice versa depending on the environmental context (Sørensen et al., 2019). Studies investigating the effects of such factors revealed that introducing abiotic stressors like increased salinity or heat shock (Duncan et al., 2010; Hori and Fujishima, 2003) can have severe consequences even in a balanced system. Changing environmental conditions such as elevated temperatures can shift the symbiont's impact toward increased virulence (defined as a reduction of host fitness) and therewith being an additional stress factor as observed for the system *Paramecium*-*Caedibacter* (Dusi et al., 2014). On the contrary, the host's stress tolerance might be boosted as observed in the symbiosis between *Paramecium* and endonuclear *Holospora undulata* (Hori et al., 2008). These infected cells show increased resistance toward osmotic shock (Fujishima, 2009b; Duncan et al., 2010) and acquire heat-shock resistance via an increased HSP70 expression (Hori and Fujishima, 2003; Fujishima et al., 2005). Increased HSP70 expression levels resulting from stable long-term infections have been reported for other symbiotic systems (Kodama et al., 2014; Grosser et al., 2018) as well.

Another advantageous trait provided by bacterial endosymbionts to *Paramecium* is the so-called killer trait (Schrallhammer et al., 2012; Koehler et al., 2019). Bacteria belonging to the genera *Caedibacter* and *Caedimonas* (Schrallhammer et al., 2018) provide their hosts with a complex strategy to outcompete congeners not harboring the respective bacterial symbiont (Schrallhammer et al., 2018). Refractile bodies (R-bodies) produced by the endosymbionts act as delivery system for an unidentified toxin which causes the death of sensitive, aposymbiotic, or symbiont-free paramecia after ingestion (Schrallhammer et al., 2012; Koehler et al., 2019).

Next to consequences for host and symbiont, symbiotic interactions have the ability to shape patterns of genetic diversity (Weiler et al., 2020), hence playing an important role for the dynamics of ecosystems with implications not only for the symbiotic partners themselves but also for other species abundant in this community (Banerji et al., 2015; Bjorbækmo et al., 2020). Exemplary, parasites infecting hosts across all trophic levels have the ability to drastically alter their host's behavior and morphology (Lafferty et al., 2008; Sukhdeo, 2010) as in the case of the parasite *H. undulata* which lead to changes in the phenotype of its host (*Paramecium*) to evade predation (Banerji et al., 2015).

Over the course of 5 years, a small lake in eastern Germany was repeatedly sampled. Paramecia were isolated, cultivated, and examined for bacterial endosymbionts implementing the full-cycle rRNA approach in order to identify the intracellular organisms. This approach allowed to detect changes in the community of intracellular bacteria over time at this ecosystem. Potential causes for the observed shifts in the endosymbionts prevalence were experimentally addressed by analysing their impact on the respective host organisms. Comparative experiments revealed the effect of infected paramecia on naturally symbiont-free or aposymbiotic strains.

## 2. Materials and Methods

### 2.1. Lake Nymphensee and Sample Collection

**Figure 1:**
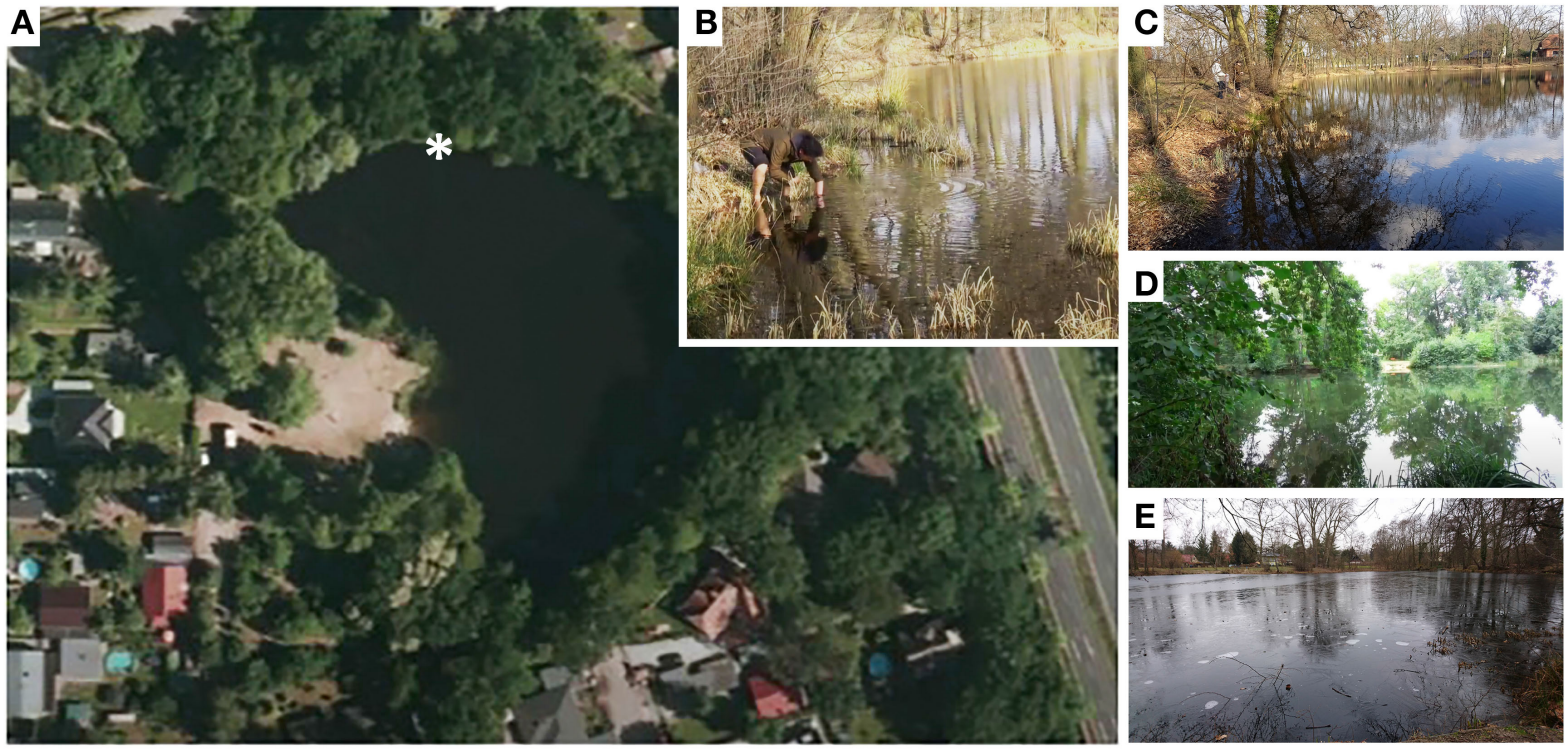
Lake Nymphensee in different seasons. **(A)** Topview of Lake Nymphensee (Brandenburg, Germany) with asterisk depicting the sampling site. **(B)** Sampling in late autumn. The lake in **(C)** spring, **(D)** summer, and **(E)** winter.

The *Paramecium* strains isolated in this study derive from Lake Nymphensee (N52 17.442, O13 27.180), a flooded former gravel pit located in Rangsdorf (Brandenburg, Germany) (Figure 1) where bathing as well as fishing are allowed. Water and sediment samples were collected from the littoral zone at 13 sampling time points over a period of 5 years (2015–2019, Supplementary Table S1). At each sampling occasion, four to six water samples, a few centimeters apart from each other, were collected from the specific littoral reed area shown in Figure 1B, covering approximately two square meters. Permanent markers like, trees and a recognizable embayment of the lakefront were used for spatial orientation throughout the seasons. Strain identifiers, e.g., RanNy1505-L1, are composed of the name of the closest village (*Ran*gsdorf), the lake (Lake *Ny*mphensee), the year (20*15*), and month (May, *05*) of sampling and a combination of numbers and letters for the specific monoclonal cell line (here *-L1*). Macroparticles like small pebbles and decomposing plant materials as well as potential *Paramecium* predators such as copepods were removed by filtering the sample over common tea filterpaper (Size 3, Profissimo, Karlsruhe, Germany) usually directly at the sampling site. One or two rice grains were added to the filtered water. These served as nutrient supply for environmental bacteria preyed upon by the ciliates. Details regarding the isolation of cells and establishment of monoclonal *Paramecium* cell lines are given in Supplementary Information S1.

### 2.2. Fluorescence *in situ* Hybridization

To confirm presence or absence as well as the identity of the bacterial endosymbionts fluorescence *in situ* hybridization (FISH) was performed (Supplementary Information S2). Used probes are detailed in Supplementary Table S2. At least 20 cells per hybridization were examined.

### 2.3. Molecular Identification of *Paramecium* Strains and Their Endosymbionts

A first identification of the newly established *Paramecium* strains was performed based on morphological criteria (Fokin, 2010). This was confirmed by applying the FISH probe PAUR588 (5′-AGCCAACTAGTAACTGACTC-3′), which discriminates between members of the *Paramecium aurelia* complex, *Paramecium jenningsi* and *Paramecium schewiakoffi* versus other *Paramecium* species. Alternatively, the cytochrome C oxidase 1 (COI) gene was used as molecular marker and amplified (see Supplementary Information S3 for details of DNA extraction) as detailed before (Barth et al., 2006). In order to confirm the affiliation to the respective *Paramecium* species, the obtained sequences were compared to available *Paramecium* COI sequences. Maximum likelihood (ML) analysis using IQ-TREE (Nguyen, 2015) was performed on an alignment (ARB program; Ludwig et al., 2004) of 165 mtCOI sequences considering 525 aligned positions. The alignment was trimmed to the length of the shortest sequence at both ends. The best-fit evolutionary model (Kalyaanamoorthy et al., 2017) following the Bayesian information criterion (BIC) is TVM+F+I+G4. Ultrafast bootstrap support with 1,000 pseudoreplicates (Hoang et al., 2018) was calculated by IQ-TREE. The 16S rRNA gene was used as molecular marker for the identification of the bacterial endosymbionts. Used primer combinations and PCR protocols are listed in Supplementary Tables S3–S6. Phylogenetic analyses of obtained sequences included 170 sequences of *Holosporales* as well as 171 sequences of *Rickettsiales* considering 1,348 and 1,328 positions, respectively. ML analyses were run using IQ-TREE, performed as described above, the applied evolutionary substitution model was TVMe+R4 (*Rickettsiales*) and TVMe+R5 (*Holosporales*).

### 2.4. Comparative Growth Experiments

The endosymbionts' effect on their hosts was determined by comparing the growth of infected cells and corresponding aposymbiotic and symbiont-free ones, respectively. Therefore, the *Paramecium biaurelia* strain RanNy1702-M5 harboring *Caedimonas varicaedens* and the genetically identical aposymbiotic strain RanNy1702-M5-AB as well as *Paramecium caudatum* RanNy1505-L4 with “*Candidatus* Megaira polyxenophila” (in the following referred to as *M. polyxenophila*) and symbiont-free RanNy1804-09 were used. In case of *C. varicaedens*, a treatment (Supplementary Information S4) with 125 μg mL^−1^ streptomycin achieved the elimination of the endosymbiont. For *M. polyxenophila*, removal of these symbionts with tetracycline as described elsewhere (Pasqualetti et al., 2020) was not successful and a symbiont-free, thus not genetically identical strain from Lake Nymphensee which was never observed to carry endosymbionts (RanNy1804-09) was included. Paramecia were adjusted to exponential growth by daily supplementation of 1/2 volume bacterized CM for 3 days. Cell densities were adjusted to approximately 100 cells mL^−1^. The cells were provided with equal volumes of bacterized CM and for each strain three replicates were included. Samples (500 μl) were taken at several time points and cell densities determined (Bella et al., 2016). In addition, FISH was performed with samples collected at the beginning, after 96 h, and at the end of the experiment after 260 h. A logistic growth model was fitted according to Dusi and colleagues (Dusi et al., 2014) using R (version 3.6.3; RStudio Team 2020) and exponential growth rate and carrying capacity were calculated. Statistically significant differences between infected and aposymbiotic or symbiont-free strains were assessed by an unpaired, two-tailed t-test with Welch's correction (GraphPad Prism, version 6, GraphPad, San Diego, USA).

### 2.5. Killer Test Assays

Killer test assays were performed as described previously (Koehler et al., 2019). In brief, R-body production by the bacterial endosymbionts was promoted by not feeding the paramecia for at least 7 days. Infection status of all used strains was verified via FISH. Three strains were tested for potential killer trait activity, i.e., *P. biaurelia* RanNy1702-M5 hosting *C. varicaedens, P. caudatum* RanNy1505-L4 infected by *M. polyxenophila*, and the known killer *Paramecium tetraurelia* 51K carrying *Caedibacter taeniospiralis* (Grosser et al., 2018) was included as control for the killer trait toxicity as described previously (Koehler et al., 2019). The susceptibility toward lethal effects was assessed for aposymbiotic *P. biaurelia* RanNy1702-M5-AB (generated by streptomycin treatment, Supplementary Information S4), symbiotic *P. caudatum* RanNy1505-L4 (used as potential killer as well as susceptible strain), and symbiont-free *P. caudatum* RanNy1804-09. All tests were performed in parallel with three replicates for each combination. In such a single combination, ten *Paramecium* cells were exposed to endosymbionts released after mechanical lysis from approximately 40 *Paramecium* cells tested for potential killer trait activity. The lysates were prepared following the protocol by Koehler et al. (2019). Survival of paramecia was checked at 2, 4, 6, 8, 18, 20, 22, and 24 h post-exposure. Potential statistically significant differences in susceptibility and lethal effect were detected by analysis of variance (ANOVA, GraphPad Prism).

## 3. Results

### 3.1. Establishment of Monoclonal *Paramecium* Strains From Lake Nymphensee and Identification of *Paramecium Species*

**Figure 2:**
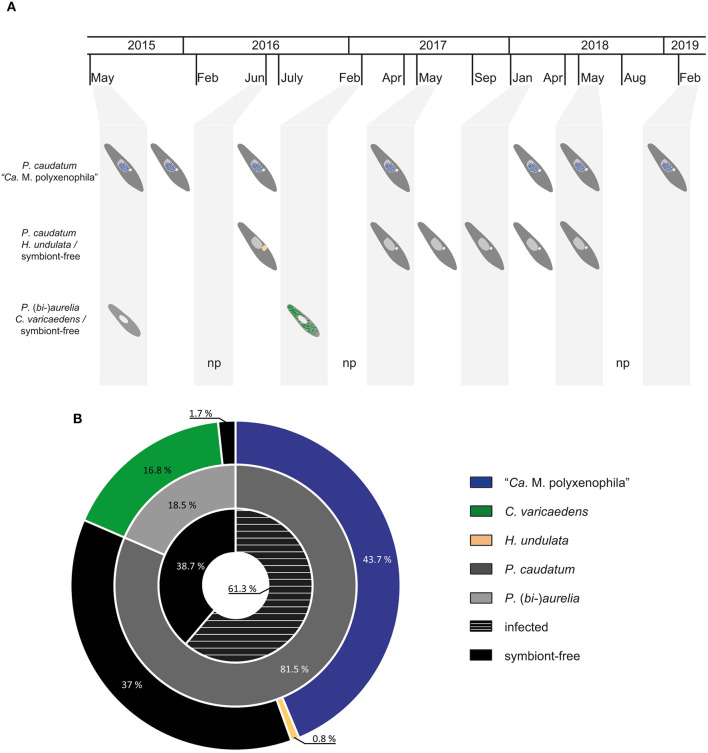
Summary of bacterial endosymbionts and their respective hosts over the course of 5 years of sampling. **(A)** Overview of paramecia (dark gray - *Paramecium caudatum*; light gray - *Paramecium (bi-)aurelia*) and their bacterial endosymbionts (blue - "*Ca*. Megaira polyxenophila", macronucleus; green - *Caedimonas varicaedens*, cytoplasm; light orange - *Holospora undulata*, enlarged micronucleus) with respect to different sampling time points at Lake Nymphensee. **(B)** Nested pie chart shows ratio between symbiont-free and infected *Paramecium* strains (black and black with white stripes, inner tier) resulting from 5 year sampling at Lake Nymphensee. The middle tier depicts relative quantities of collected *P. caudatum* and *P. (bi-)aurelia* while the outer tier shows relative prevalence of bacterial endosymbiont with respect to their specific host. Sampled *Paramecium bursaria* are not indicated. Time points with no paramecia in the screened water samples are marked with “np”. “*Ca*.” stands for “*Candidatus*”.

Over the course of 5 years of repeated sampling at Lake Nymphensee, water samples were collected at 13 different time points (Figure 2A). From all samples that arrived at the laboratory and contained living ciliates, monoclonal *Paramecium* sp. strains were established and further characterized (Supplementary Table S1). In a few samples (RanNy1606, RanNy1704, and RanNy1808), neither living paramecia nor other ciliates were detected. Typically, these were samples which had either not been filtered directly at the sampling site and contained high numbers of copepods and other micropredators (dead or alive) or which experienced an exceptional long shipping time (more than 3 days). In total, 119 monoclonal strains from ten sampling occasions were screened for the presence of intracellular bacteria and assigned to a *Paramecium* species. A quick species assignment based on observation of morphological characters revealed that *P. caudatum, P. bursaria*, and members of the morphologically indistinguishable *P. aurelia* complex occurred in Lake Nymphensee. The most frequently observed species was *P. caudatum* (Figure 2B), monoclonal strains were established from nine out of ten sampling time points (exception: February 2017). *P. aurelia* and green *P. bursaria* harboring intracellular algae (Flemming et al., 2020) were detected in two cases (May 2015 and February 2017, respectively, July 2016 and May 2017) and isolated as well. The strains established from these samplings showed no bacterial infections (data not shown). In order to refine species identification of infected strains, COI gene sequences were obtained and used as molecular marker in phylogenetic analysis (Supplementary Figure S1). For each sampling time point, a minimum of three to five representative COI sequences were obtained. For September 2017 and January 2018, the isolated strains could only be maintained briefly under laboratory conditions preventing the COI gene sequences to be obtained. Hence, they have not been included in the phylogenetic analysis. Detailed assignment of established strains is shown in Supplementary Table S1. COI sequences of 10 strains isolated from the sample collected in February 2017 (RanNy1702) affiliated with other *P. biaurelia* sequences. All other obtained sequences (43) deriving from six other sampling time points (Supplementary Table S1) cluster with *P. caudatum* strains. COI sequences are deposited at Genbank with accession numbers MW562072-MW562126. Results of the phylogenetic analysis are in good agreement with morphological observations, i.e., all *P. caudatum* strains were confirmed and members of the *P. aurelia* complex were identified as *P. biaurelia* strains.

### 3.2. Detection and Identification of Bacterial Endosymbionts

**Figure 3:**
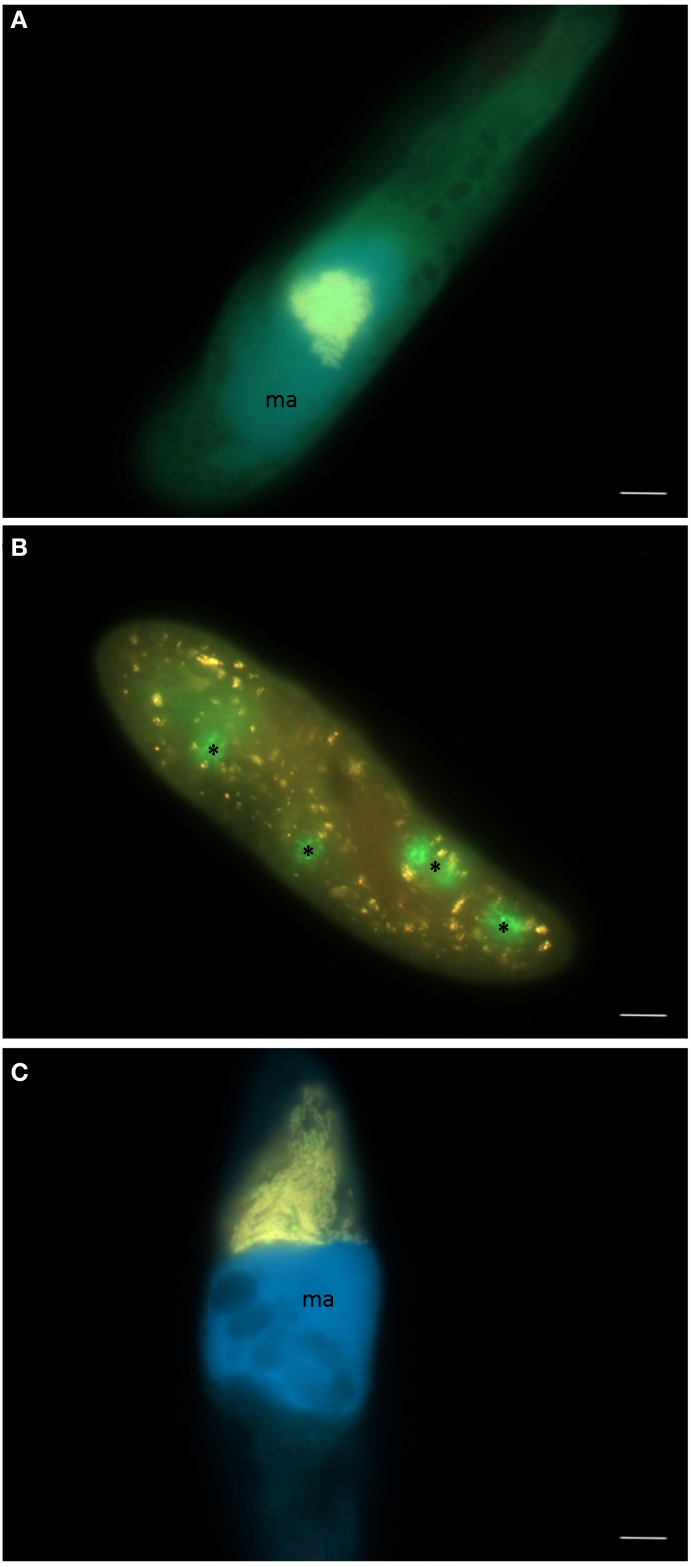
Three different endosymbiotic bacteria observed in paramecia isolated from Lake Nymphensee, Germany. **(A)** “*Candidatus* Megaira polyxenophila” in the macronucleus of *Paramecium caudatum* RanNy1505-L4, **(B)** *Caedimonas varicaedens* in the cytoplasm of *Paramecium biaurelia* RanNy1702-M5 and **(C)** *Holospora undulata* in the micronucleus of *Paramecium caudatum* RanNy1607-15. **(A–C)** Merge of signals from FISH applying the universal probe EUB338 (green signal), a second probe (red signal) specific for species-level (CC23a, **B**), respectively, genus-level (Megenus-487, **A**; Holosp1, **C**), and DAPI (blue signal) visualizing the macronucleus (ma). Asterisks indicate food vacuoles filled with the food bacterium *Raoultella planticola*. Scale bars = 10 μm.

**Figure 4:**
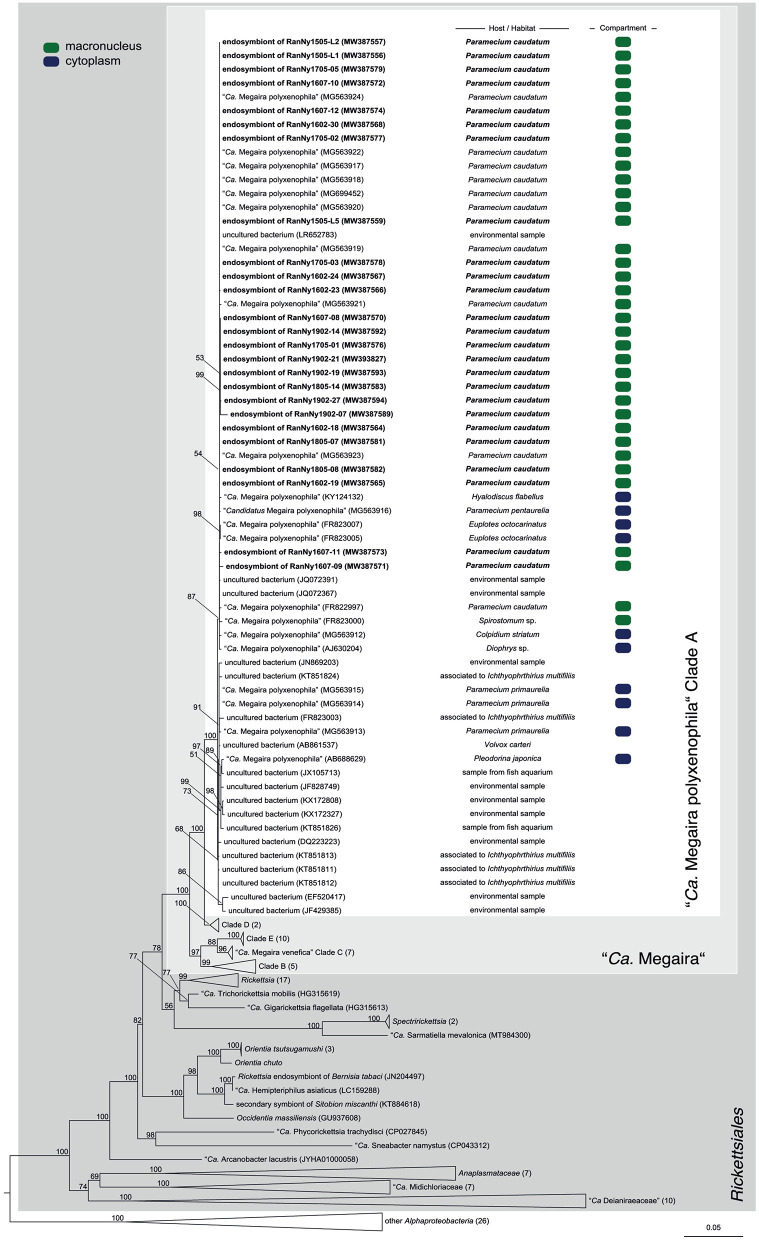
Phylogenetic reconstruction of the order *Rickettsiales* indicating the positioning of the newly characterized bacterial endosymbiont's sequences. Maximum likelihood tree calculated with IQ-TREE based on an alignment of 171 16S rRNA gene sequences. The applied best-fit evolutionary model is TVMe+R4. Node labels indicate ultra fast bootstrap support values of IQ-TREE, shown if equal or higher than 50%. Occupied host compartment (if data available) is indicated by colored blocks (green: macronucleus; blue: cytoplasm). Newly characterized sequences are shown in bold. Trapezoid forms represent collapsed sequence groups, numbers in brackets indicate the number of collapsed sequences included. Scale bar represents 0.05 nucleotide substitutions per site. “*Ca*.” stands for “*Candidatus*.”

**Figure 5:**
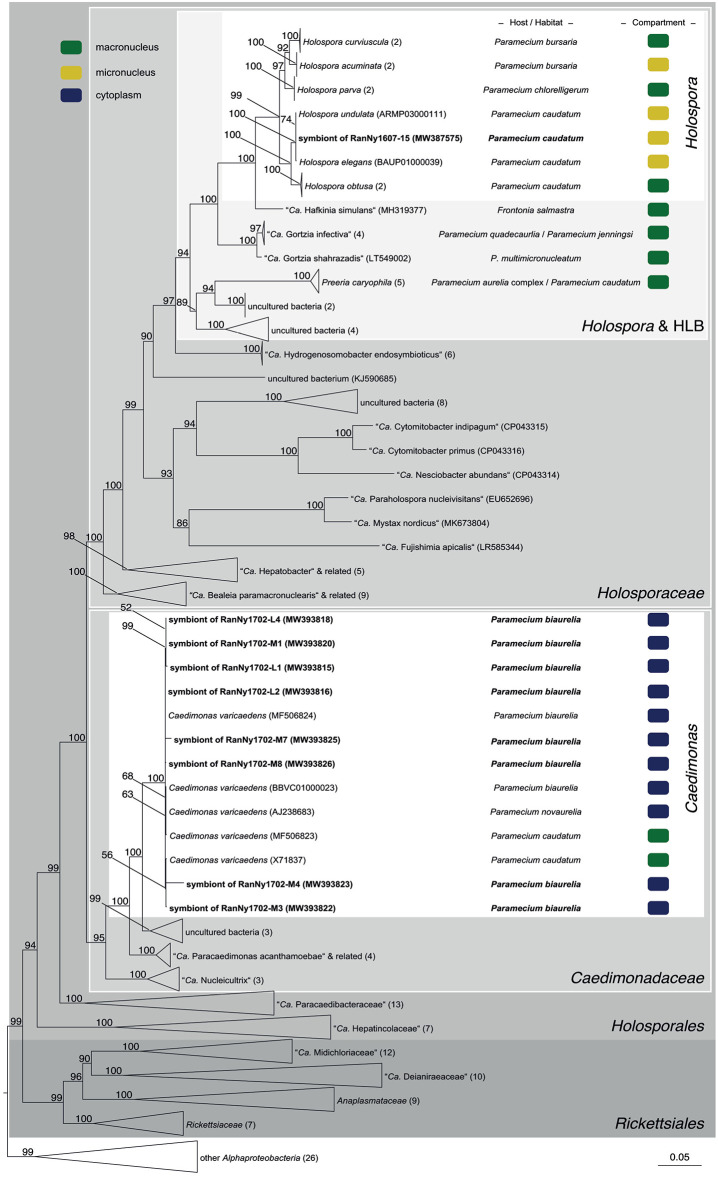
Phylogenetic reconstruction of the order *Holosporales* indicating the positioning of the newly characterized bacterial endosymbiont's sequences. Maximum likelihood tree calculated with IQ-TREE based on an alignment of 170 16S rRNA gene sequences. The applied best-fit evolutionary model is TVMe+R5. Node labels indicate ultra fast bootstrap support values of IQ-TREE, shown if equal or higher than 50%. Occupied host compartment (if data available) is indicated by colored blocks (yellow: micronucleus; green: macronucleus; blue: cytoplasm). Newly characterized sequences are shown in bold. Trapezoid forms represent collapsed sequence groups, numbers in brackets indicate the number of collapsed sequences included. Scale bar represents 0.05 nucleotide substitutions per site. “*Ca*.” stands for “*Candidatus*.”

In total, 119 monoclonal *Paramecium* strains were screened for the presence or absence of bacterial endosymbionts via FISH. The observed pattern of infection, respectively, symbiont-free status and the prevalence of symbionts within the strains established from a single sampling event were complex (Figure 2). In brief, the established strains can be grouped in four categories. Of 119 screened strains (i) 43.7% carried an infection present in the macronucleus, (ii) 16.8% in the cytoplasm, and (iii) 0.8% in the micronucleus. The remaining strains (38.7%) were categorized as (iv) symbiont-free (Figures 2A,B). Cases of double infection were never observed. The full-cycle rRNA approach was followed to characterize in total 60 bacterial endosymbionts in detail, sequencing the 16S rRNA gene and confirming the results by FISH (Figure 3). The remaining strains were only examined via FISH. Preliminary phylogenies inferred on the basis of 16S rRNA gene sequences revealed that sequences obtained from macronuclear infections affiliate to the type strain of *M. polyxenophila* (accession number AJ630204) and related sequences. In total, 42 endosymbiont strains from Lake Nymphensee cluster together with other representatives of *M. polyxenophila* (Figure 4) and share 99.47–99.77% sequence similarity with the type strain. Thus, they are well above the 98.65% threshold for species discrimination (Kim et al., 2014). In case of the cytoplasmic and micronuclear infections, 20 almost full-length 16S rRNA sequences were obtained. Ninety sequences associate in phylogenetic reconstructions with the cytoplasmic endosymbiont *C. varicaedens* (BBVC01000023) and other members of this species (Figure 5). These findings were confirmed via FISH using the species-specific probe CC23a (Figure 3B). All sequences share 99.26–99.93% similarity with the type strain. The exception is strain RanNy1702-M4 with only 98.15% sequence similarity. The remaining sequence was obtained from the only *Paramecium* strain harboring a mirconuclear infection, RanNy1607-15. This 16S rRNA gene sequence affiliates with *H. undulata* (Figure 5). High bootstrap support as well as 99.93% sequence similarity to the type strain (ARMP03000111) confirm the species affiliation. FISH revealed a hyperinfection in the enlarged micronucleus of this strain (Figure 3C).

### 3.3. Impact of *M. polyxenophila* and *C. varicaedens* on Their Host's Growth

**Figure 6:**
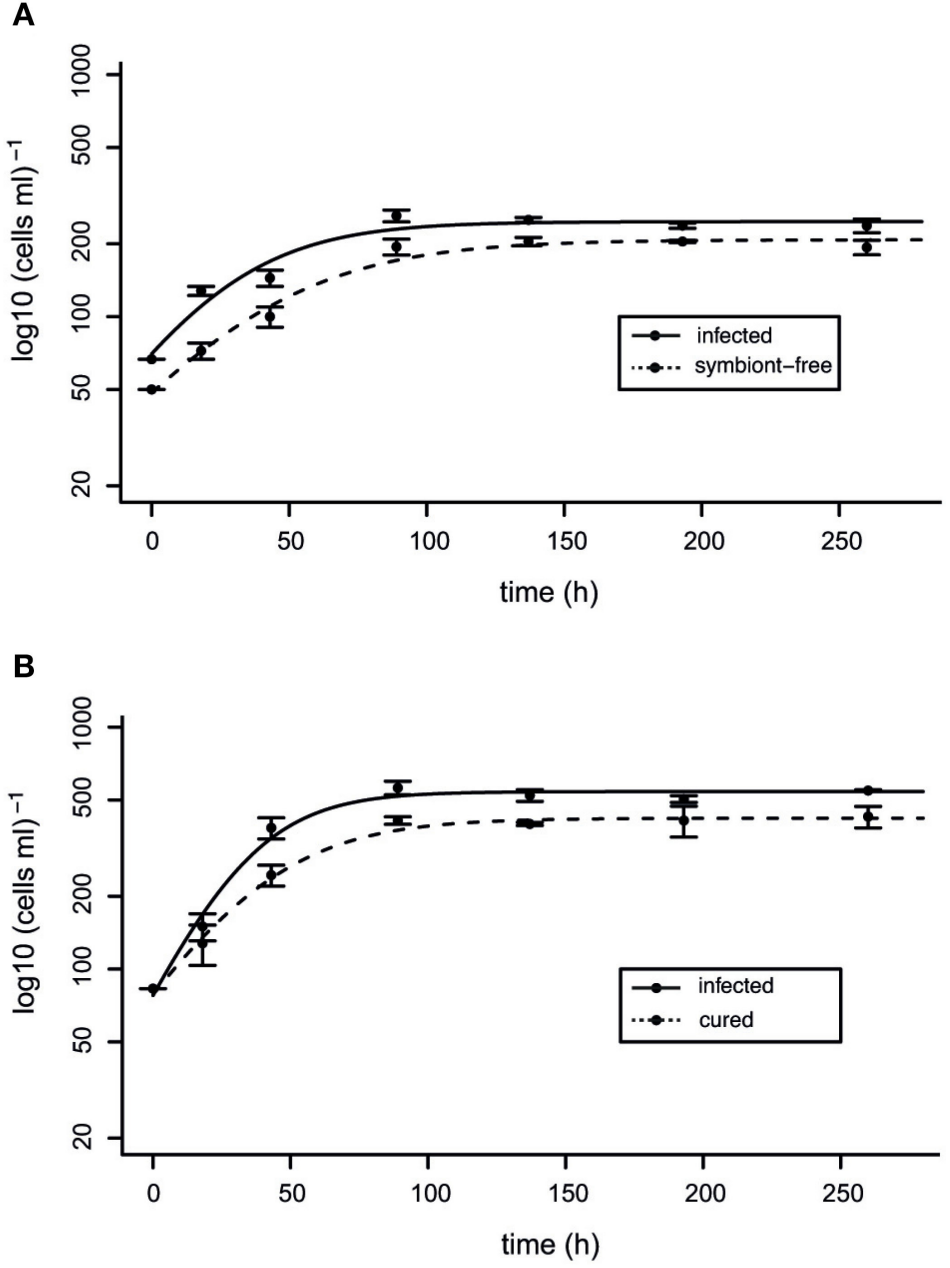
Fitness impact of RanNy endosymbionts. Comparison between the population growth of infected and cured or symbiont-free *Paramecium* cells. **(A)** *Paramecium caudatum* RanNy1505-L4 harboring “*Candidatus* Megaira polyxenophila” (solid line) and symbiont-free *P. caudatum* RanNy1804-09 (dashed line). **(B)** *Paramecium biaurelia* RanNy1702-M5 harboring *Caedimonas varicaedens* (solid line) and the corresponding cured cell line RanNy1702-M5-AB (dashed line). Depicted are mean cell densities mL^−1^ of three replicates ± SD at different time points (in hours). A logistic regression model fits the regression (lines).

A comparative growth assay was performed in order to investigate the impact of the bacterial endosymbionts *M. polyxenophila* and *C. varicaedens* on their respective hosts' fitness (Figure 6). Prior to the experiment, infection status for all included strains was confirmed via FISH (Supplementary Figure S2). Therefore, the growth of *P. caudatum* RanNy1505-L4 infected with *M. polyxenophila* and symbiont-free *P. caudatum* RanNy1804-09 was monitored over a period of 260 h. The latter strain was included in the experiments since elimination of the bacterial endosymbiont of RanNy1505-L4 using tetracycline was not successful. Additionally, the *C. varicaedens* infected strain *P. biaurelia* RanNy1702-M5 and the genetically identical but from the infection successfully cured strain RanNy1702-M5-AB were compared. All four strains, independent of infection status, reached their carrying capacity after approximately 100 h, whereas both infected strains exhibit higher cell densities compared to their aposymbiotic or symbiont-free counterparts. Statistically significant differences (*p* ≤ 0.05) were only observed between the carrying capacity of symbiont-harboring and, respectively, cured or symbiont-free strains (Supplementary Figure S3).

### 3.4. Susceptibility and Killer Potential of Paramecia and Their Endosymbionts Isolated From Lake Nymphensee

**Figure 7:**
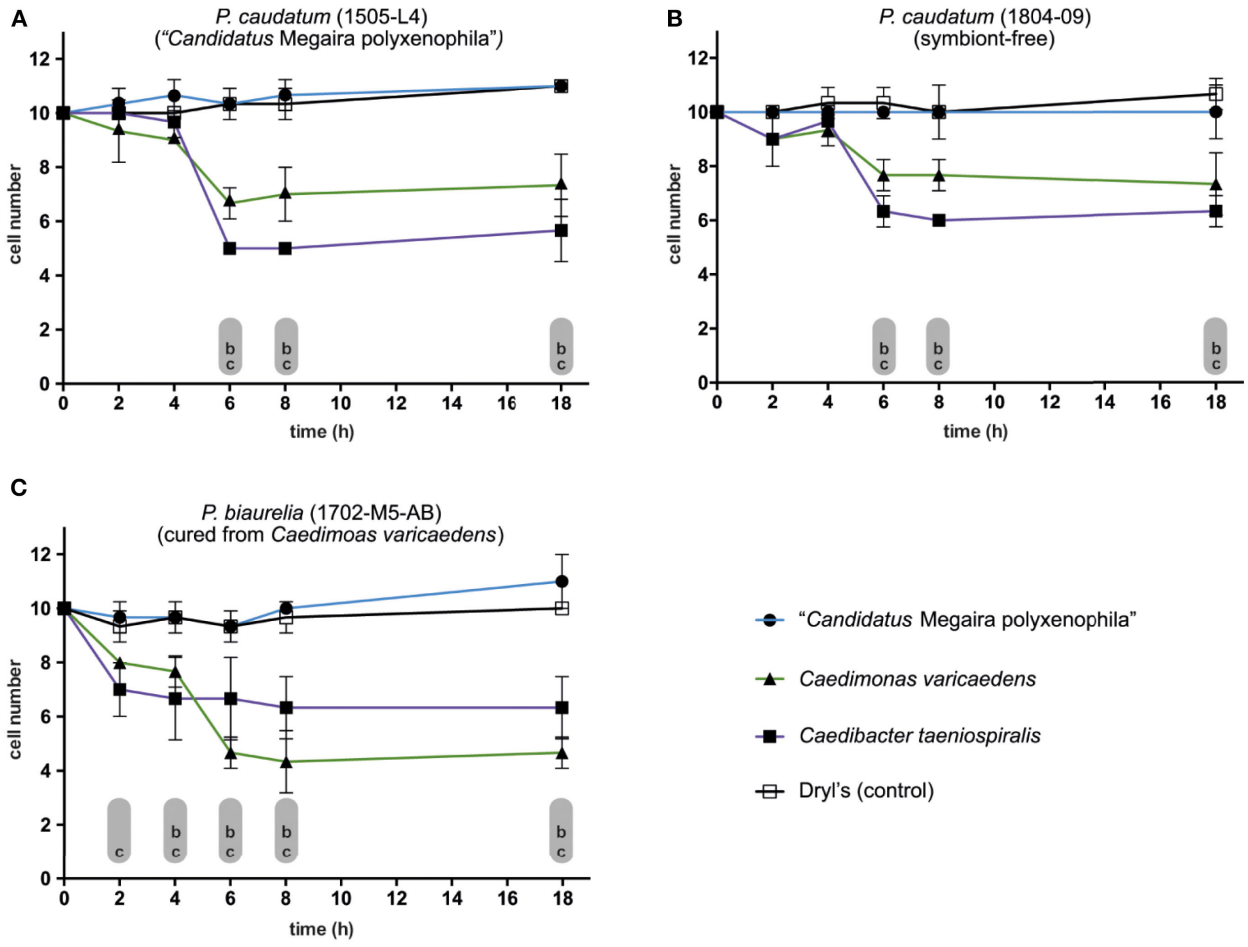
Susceptibility of different *Paramecium* strains toward potential killer trait toxicity. **(A–C)** Monitoring the survival of exposed (sensitive) cells to bacterial endosymbionts released via mechanical lysis from infected *Paramecium* strains (lysate of killer cells). Exposure of **(A)** *Paramecium caudatum* RanNy1505-L4 (harboring “*Candidatus* Megaira polyxenophila”, in the following *M. polyxenophila*), **(B)** *P. caudatum* RanNy1804-09 (symbiont-free) and **(C)** *Paramecium biaurelia* RanNy1702-M5-AB cured from *Caedimonas varicaedens* to lysates obtained from three different symbiont-bearing paramecia (solid symbols) or Dryl's solution as control (empty square). Released bacteria in the lysates were *M. polyxenophila* (from *P. caudatum* RanNy1505-L4; blue line, solid circle), *C. varicaedens* (from *P. biaurelia* RanNy1702-M5; green line, solid triangle) and *Caedibacter taeniospiralis* (from *Paramecium tetraurelia* 51K; violet line, solid square). A RM two-way ANOVA followed by a multiple comparisons test (Dunnett's correction) was used for statistical analysis (*p* ≤ 0.05). The mean number (three replicates) of surviving cells during exposure to one of the lysates was compared to the control (lowercases indicate statistically significant differences therein; comparison between susceptibility toward control and (a) *M. polyxenophila*, (b) *C. varicaedens*, and (c) *Caedib. taeniospiralis*).

*C. varicaedens*, known to provide its host the ability to kill symbiont-free congeners was detected in *P*. (*bi*-)*aurelia* strains isolated in February 2017 (RanNy1702; detailed information about strains is given in Supplementary Table S1). This trait is also found in the better known *Caedib. taeniospiralis*. Hence, some of the established strains were assessed for killer trait toxicity as well as for susceptibility toward the killer trait. Cells from all tested strains showed a significant decrease in numbers when exposed to released *Caedib. taeniospiralis* 51K but remained at a constant level at lysate-free conditions (Figures 7A–C). After 6 h of exposure, a clear lethal impact of released *Caedib. taeniospiralis* and *C. varicaedens* was observed in *P. caudatum* carrying *M. polyxenophila* from Lake Nymphensee. A similar effect, albeit less pronounced, was observed for the other tested but symbiont-free *P. caudatum* strain (Figure 7B). *P. biaurelia* cured from *C. varicaedens* (Figure 7C) showed a significant decrease in cell numbers early after exposure to released symbionts, starting from 2, respectively, 4 h (Figure 7C). Interestingly, the strongest lethal effect was observed when the cells were challenged with their previous endosymbionts (reduction by 50–60%). As for the above mentioned strains, 8 h after exposure the maximal reduction in cell number was reached.

Interpreting those data from another perspective, namely with respect to lethal potential of the released lysates, it showed that *M. polyxenophila* did not induce a decrease in any of the tested strains. However, both *C. varicaedens* and *Caedib. taeniospiralis* caused statistically significant cell number reduction in each exposed strain. For the Nymphensee symbiont *C. varicaedens*, its effective cell number reduction varied between 20% in the symbiont-free *P. caudatum* strain to 50–60% in the *P. biaurelia* strain genetically identical to its own host but lacking the endosymbiont (Supplementary Figure S5).

## 4. Discussion

### 4.1. Temporal Persistence of *M. polyxenophila* at Lake Nymphensee Over a 5 Year Period

During the 5 year sampling period, we observed shifts in the occurrence of *Paramecium* species and in the prevalence of three bacterial endosymbionts. While the dependence of the obligate intracellular bacteria on suitable *Paramecium* hosts is obvious, the possible effects of symbionts on species composition and interactions is in most cases not understood. Several causes (and combinations thereof) for our observations are possible. Here, we addressed the impact of bacterial endosymbionts on their hosts' fitness and on competition between infected and symbiont-free paramecia as possible causes for changes in the prevalence of infected paramecia. Alternative or additional scenarios include differences in infectivity and host range of the intracellular bacteria. *P. caudatum* dominates the collected water samples. Only at two occasions we isolated members of the *P. aurelia* species complex which were identified as *P. biaurelia* for February 2017. The third observed species was *P. bursaria*. It harbored the green algae *Micractinium conductrix* (Flemming et al., 2020) but bacterial endosymbionts were never observed (data not shown). Initially we observed a stable presence of *M. polyxenophila* in the dominating *P. caudatum* from May 2015 till July 2016. Noteworthy, in the only sample from which two different *Paramecium* species were isolated, namely a member of *P. biaurelia* alongside *P. caudatum*, only the latter was infected albeit this endosymbiont's host range covers several *Paramecium* species. *M. polyxenophila* can infect the cytoplasm of different *P. aurelia* species, i.e., *Paramecium primaurelia* and *Paramecium pentaurelia* (Lanzoni et al., 2019; Pasqualetti et al., 2020), hence, exhibiting a wide interaction spectrum presumably due to characteristics in host cell physiology (Potekhin et al., 2018). However, an infection of *P. biaurelia* with *M. polyxenophila* has not been observed so far. This might be explained by potential strain specificity or possibly even resistance since the latter acts as key determinant in parasitic host-symbiont interactions as demonstrated for *P. caudatum* and *H. undulata* (Weiler et al., 2020). The dominating prevalence of *M. polyxenophila* in the macronucleus in *P. caudatum* is intermitted by the appearance of *H. undulata* and *C. varicaedens*. In July 2016, *H. undulata* was detected next to *M. polyxenophila* as a second endosymbiont albeit with a much lower prevalence in the examined strains and the symbionts were not observed simultaneously infecting one host cell. *H. undulata* is typically considered a parasite living in the micronucleus of *P. caudatum* using nucleotides or ribonucleotides from its host as energy source (Garushyants et al., 2018), exhibiting very strict host and compartment specificity (Schrallhammer and Potekhin, 2020). Next to the high costs inflicted on their hosts, this parasite is known for its highly effective horizontal transmission (Fokin and Görtz, 2009; Magalon et al., 2010). Thus, *H. undulata* might have been observed at the beginning of an infection outbreak. It is unclear whether this bacterium can infect only the otherwise undetected minority of symbiont-free *P. caudatum* cells in the respective population or if it replaces the macronuclear *M. polyxenophila*. Paramecia can lose or even actively expel their symbionts under high stress conditions like temperature stress (Dusi et al., 2014), but a symbiont-induced replacement has not been caught in the act so far. Nevertheless, in the subsequent sampling after the appearance of *H. undulata*, no *P. caudatum* were detected. This could have been caused by detrimental effects of *H. undulata* but also the R-body producing killer symbiont *C. varicaedens* appearing in strains from the subsequently collected water sample in February 2017. No additional *Paramecium* species were detected simultaneously. Even though the detected endosymbionts *M. polyxenophila* and *C. varicaedens* have been reported from the respective other host species (Schrallhammer et al., 2018; Landmann, 2019), we did not observe any such host shift in the strains investigated from Lake Nymphensee. *Holospora* species have been described co-occurring with other bacteria (Potekhin et al., 2018) but although we detected it in the same water sample and host as *M. polyxenophila*, no simultaneous prevalence in the host cell in the respective analyzed samples was observed. High similarities in the 16S rRNA gene sequence (99–100%) between phylogenetically and geographically distant *Paramecium* hosts have been considered as indication for horizontal transmission capabilities of those symbionts (Schrallhammer et al., 2013; Pasqualetti et al., 2020), albeit such infection experiments under laboratory conditions were unsuccessful (Lanzoni et al., 2019) so far. Further impairments for addressing the nature of the symbiotic association of *M. polyxenophila* and e.g., *P. caudatum* are the obligate intracellular life style of this bacterium (Castelli et al., 2016) as well as the difficulties to eliminate the symbiont (Pasqualetti et al., 2020).

Bacteria belonging to the genera *Caedibacter* and *Caedimonas* provide their host with a complex, costly strategy to outcompete symbiont-free congeners (Schrallhammer et al., 2018). The susceptibility of *M. polyxenophila* harboring and symbiont-free *P. caudatum* toward the killer trait was tested revealing a susceptibility of all *P. caudatum* RanNy strains toward the lethal toxins released by *C. varicaedens*. Furthermore, neither a negative effect of *M. polyxenophila* on host fitness nor an increase in host resistance compared to symbiont-free cells was observed. Thus, *C. varicaedens* can provide its *P. biaurelia* host a competitive advantage if co-occurring with infected or symbiont-free *P. caudatum*.

Despite this selective advantage and even though no negative effect on host fitness was observed, *C. varicaedens* harboring paramecia were not detected in subsequent samplings. In this study, we focused on the bacterial endosymbiont's influence on host fitness and on competition between symbiont-harboring and symbiont-free paramecia as potential explanation for the observed shifts in prevalence in this specific location. Due to the advantageous killer trait mediated by *C. varicaedens, P. biaurelia* collected in February 2017 would have been assumed to persist over subsequent sampling events which is not supported by our data. Previous studies highlighted biotic and abiotic factors with the potential to strongly affect ciliate species composition in lake ecosystems (Przyboś et al., 2011; Sonntag et al., 2006). For example, spatial localization in the aquatic environment has been considered a highly effective parameter shaping ciliate diversity and composition (Sonntag et al., 2006; Babko et al., 2020). Infectiousness as well as interspecific competition were additionally addressed as a possible explanation for the disappearance of *C. varicaedens* despite the competitive advantage provided. Different studies showed that very low host division rates of the *Paramecium* host caused by conditions such as food depletion can result in *C. varicaedens* overgrowth. A scenario in which the otherwise mutualistic nature of this symbiotic association shifts toward parasitism, ultimately resulting in the hosts's subsequent death (Kusch et al., 2002; Schmidt et al., 1987). Consequently, additional biotic as well abiotic factors likely played a role in the disappearance of *P. biaurelia* at Lake Nymphensee over the course of 5 years.

Starting from May 2017, *M. polyxenophila* begins to infrequently re-appear in cells from the analyzed water samples accompanied by symbiont-free *P. caudatum* until May 2018. Hereinafter, this symbiont rebuilds to 100% prevalence till the end of our sampling in February 2019. A likely explanation for this “recovery” and temporal persistence of *M. polyxenophila* infected *P. caudatum* is the observed positive effect of this symbiont on its host. Comparative growth assays revealed that *M. polyxenophila* harboring *P. caudatum* showed an increased cell density in comparison to their symbiont-free counterparts. In support of this finding are results demonstrating that *M. polyxenophila* has no obvious negative impact on its host's fitness but conditionally mutualistic effects (Pasqualetti et al., 2020). Consequently, *M. polyxenophila* provides an indirect competitive advantage over symbiont-free *P. caudatum* cells potentially resulting in the observed shift from symbiont-free to symbiont-harboring paramecia over time.

## 5. Conclusion

This study addresses factors influencing occurrence and temporal persistence of bacterial endosymbionts in natural paramecia populations isolated from a small lake ecosystem over a time span of 5 years. Competition and symbionts' fitness impacts are assessed as important factors explaining the observed dynamics in the endosymbionts' occurrence. Our observations indicate for the examined system that positive effects on host fitness trump negative impact on host competitors in natural habitats in the long-term. Endosymbiotic interactions between bacteria and eukaryotes are important, intensely studied aspects in ecology and evolution. These interactions between ciliate hosts and their bacterial and algal (endo-)symbionts include a remarkable diversity of organisms involved. The endosymbiont's impact on their ciliate host's prevalence in e.g., lake ecosystems is one of the potentially crucial factors in predicting patterns of presence or absence of certain ciliates. Studies of infection, prevalence, and effect of bacterial endosymbionts in ciliates might be of relevance beyond their impact on ciliate fitness and frequencies. As these symbionts in total but also in particular are not restricted to highly virulent or infectious organisms as usually the case in zoonotic pathogens of medical or economic relevance, we have the possibility to gain a more holistic view of how those intracellular bacteria shape the fitness and appearance of their hosts in ecosystems.

## Data Availability Statement

The datasets presented in this study can be found in online repositories. The names of the repository/repositories and accession number(s) can be found in the article/Supplementary Material.

## Author Contributions

MS designed the research. KG organized and carried out the sampling. FF performed the experiments and conducted statistical and phylogenetic analyses. MS and FF interpreted the results. The manuscript was written by FF and MS. All authors contributed to the article and approved the submitted version.

## Funding

This research was supported by the Wissenschaftliche Gesellschaft Freiburg im Breisgau, the Grünewald-Zuberbier Foundation, and by the Research Innovation Fund of the University of Freiburg (Project 2100297401). The article processing charge was funded by the Baden-Württemberg Ministry of Science, Research and Art and the University of Freiburg in the funding programme Open Access Publishing.

## Conflict of Interest

The authors declare that the research was conducted in the absence of any commercial or financial relationships that could be construed as a potential conflict of interest.

## Publisher's Note

All claims expressed in this article are solely those of the authors and do not necessarily represent those of their affiliated organizations, or those of the publisher, the editors and the reviewers. Any product that may be evaluated in this article, or claim that may be made by its manufacturer, is not guaranteed or endorsed by the publisher.
