# Supplementary material for: Natural Shifts in Endosymbionts' Occurrence and Relative Frequency in Their Ciliate Host Population
Source: Front Microbiol. 2022 Jan 11;12:791615. doi: 10.3389/fmicb.2021.791615 (PMC8787144; doi:10.3389/fmicb.2021.791615)
Supplement: Supplementary file 1 [file Data_sheet_1.pdf]

Supplementary material

Supplementary Information S1: Media preparation and cultivation of bacteria and paramecia
Supplementary Information S2: Fluorescence *in situ* hybridization
Supplementary Information S3: DNA extraction and amplification of molecular marker genes
Supplementary Information S4: Antibiotic treatment for endosymbiont elimination

Supplementary Table S1: Established monoclonal *Paramecium* strains collected at different time points from Lake Nymphensee.
Supplementary Table S2: Probes used for characterization of bacterial endosymbionts via fluorescence *in situ* hybridization (FISH).
Supplementary Table S3: Oligonucleotides used for molecular characterization of both *Paramecium* and endosymbiotic bacteria.
Supplementary Table S4: Oligonucleotides used for sequencing of the cytochrome C oxidase I gene of paramecia and the 16S rRNA gene for bacterial endosymbionts.
Supplementary Table S5: Primer pairs used for amplification of bacterial 16S rRNA gene.
Supplementary Table S6: PCR program used for amplification of the bacterial 16S rRNA gene.

Supplementary Figure S1: Molecular phylogeny of the genus *Paramecium* based on COI gene sequences.
Supplementary Figure S2: Fluorescence *in situ* hybridization of *Paramecium* strains used in the competitive growth experiment to confirm their infection status.
Supplementary Figure S3: Comparison of the carrying capacities K and growth rates r between the infected and symbiont-free or cured *Paramecium* strains used in the competitive growth experiment.
Supplementary Figure S4: Fluorescence *in situ* hybridization of infected ("killer") and symbiont-free or aposymbiotic ("sensitives") *Paramecium* strains.
Supplementary Figure S5: Lethal potential of „*Ca*. Megaira polyxenophila", *Caedimonas varicaedens* and *Caedibacter taeniospiralis*.

Supplementary Information 1: Media preparation and cultivation of bacteria and paramecia
0.25 % Cerophyll medium (CM; Bella et al., 2016) was prepared from wheat grass powder (GSE-Vertrieb, Saarbrücken, Germany) with 500 µg L^{-1} stigmasterol (Sigma-Aldrich, Munich, Germany). It was inoculated with the bacterium *Raoultella planticola* DMSZ 3069 and incubated at 20°C with shaking at 95 rpm for at least two and a maximum of seven days prior to feeding of paramecia. Therefore, a volume of 1/10 to 1/4 was added once a week.
Volvic water (Danone Waters, Frankfurt/Main, Germany) or Dryl's solution (0.1 M Na_2HPO_4, 0.1 M NaH_2PO_4, 0.1 M $CaCl_2$, 0.1 M sodium citrate, pH 6.8) were used for washing of *Paramecium* cells or dilution of cultures. For some experiments, exhausted CM (exCM) was used. Therefore, bacterized CM which was inoculated at least seven days ago was centrifuged for 15 minutes at 4,500 x g. The supernatant was transferred to a new tube and centrifuged again. This step was repeated twice and the supernatant was filtered (0.22 µm pore filter, Macherey-Nagel, Düren, Germany). ExCM was stored at 4 °C.

Supplementary Information 2: Fluorescence *in situ* hybridization
Paramecia were washed three times and fixed with 2% w/v paraformaldehyde on microscope slides (Superfrost UltraPlus, ThermoScientific, Waltham, USA) followed by an ethanol gradient (50 %, 80 % and 100 %, 10 min each). Hybridization buffers (900 mM l^{-1} NaCl, 20 mM l^{-1} TrisHCl, 0.01% SDS) contained formamide in the range from 0-25% following the recommended concentrations specified for the used probes at 46°C (ca. 18 h). Excess probes were removed by washing (900 mM to 102 mM l^{-1} NaCl, 20 mM l^{-1} TrisHCl, 0.01% SDS, for formamide concentrations higher than 20%: additionally 5 mM EDTA) for 20 min at 48°C. After air-drying, the slides were mounted with Citifluor AF1 or Citifluor AF1-DAPI (Citifluor, London, United Kingdom).
Slides were observed by fluorescence microscopy (Axio M2, Zeiss, Oberkochen, Germany) applying the respective filter sets for 6-carboxyfluorescein (EX BP 470/40 | EM BP 525/50), Cyanine 3 (EX BP 545/25 | EM BP 605/70) and if applicable DAPI (EX BP 365 | EM BP 445/50).

Supplementary Information 3: DNA extraction and amplification of molecular marker genes

Prior to DNA extraction using the NucleoSpin Plant II Kit (Macherey-Nagel, Düren, Germany), the respective *Paramecium* cells were not fed for a minimum of eight days. To further reduce the number of extracellular bacteria the paramecia were washed repeatedly (up to twelve times) in Volvic water respectively Dryl's solution, in some cases containing 50 µg mL^{-1} streptomycin (Carl Roth, Karlsruhe, Germany), with an overnight incubation in the last washing step. This process was repeated twice. 80-200 of the so-treated ciliates were washed again three times (always without antibiotics) and subsequently fixed in 70 % ethanol. All polymerase chain reactions (PCR) were performed using a PeqStar 2× thermocycler (Peqlab Biotechnologies, Erlangen, Germany) with TaKaRa reagents and TaKaRa ExTaq polymerase (TaKaRa, Otsu, Japan). Purified PCR products (QIAquick PCR Purification Kit, QIAgen, Hilden, Germany) were sequenced at Eurofins Genomics (Eurofins, Ebersberg, Germany).

Supplementary Information 4: Antibiotic treatment for endosymbiont elimination

Caedimonas varicaedens infected *Paramecium biaurelia* RanNy1702-M5 were not fed for five to seven days. 30-50 individual cells were washed three times and transferred in 500 µl exCM with streptomycin (125 µg mL^{-1}) and incubated overnight at 24°C. This was repeated and then followed by washing in Volvic to remove residues of streptomycin and addition of bacterized CM. After two to three days of recovery, the cells were treated for a second time as described using 100 µg mL^{-1} streptomycin.

In case of "*Ca*. Megaira polyxenophila", we followed the protocol by Pasqualetti and colleagues (Pasqualetti *et al*., 2020). As the used strain *Paramecium caudatum* RanNy1505-L4 quickly showed antagonistic reactions to the recommended concentration of tetracycline (130 µg mL^{-1}), we additionally tested lower concentrations (50, 75 and 100 µg mL^{-1}). Treatment was unsuccessful in all attempts and had either no effect on the number of intracellular bacteria (low concentrations) or resulted in the death of paramecia (data not shown).

Supplementary Table S1-1: Established monoclonal *Paramecium* strains collected at different time points from Lake Nymphensee. For time points where *Paramecium* strains infected with bacterial endosymbionts were isolated, only those are listed where either symbiont and/or host have been characterized.
* representative strains included in phylogenetic analyses shown in this study; ** bacterial identification based on combination of FISH results (e.g. probes Rick527, Alf19b, Megenus), morphology, and occupied host compartment; *** indicates host identification based on morphological characters; + full cycle rRNA approach completed;
AP: Astrid Petersen; BS: Birgit Süssenbach; CS: Constanze Schindler; DH: Dilara Hasavci; JB: Jessica Bejrit; JJ: Jaqueline Jauch; JS: Johanna Schell; NH: Nis Hansen; NS: Nils Stappert; SG: Selenja Gerbi; SSf: Sophie Schäfer; SSw: Sabine Schwenk; SSz: Sabine Schwarzer.

Strain	Symbiont	Host	Full cycle rRNA approach	Symbiont ID	by	Host ID	by
RanNy1505-L1	*M. polyxenophila*	*P. caudatum*	+	16S *	NS	COI *	NS
RanNy1505-L2	*M. polyxenophila*	*P. caudatum*	+	16S *	NS	***	NS
RanNy1505-L4	*M. polyxenophila*	*P. caudatum*	+	16S	JJ	COI *	AP
RanNy1505-L5	*M. polyxenophila*	*P. caudatum*	+	16S *	SSw	COI *	SS
RanNy1505-L6	*M. polyxenophila*	*P. caudatum*	+	16S	JJ	COI	AP
RanNy1505-S1	symbiont-free	*P. aurelia*				***	NS
RanNy1505-S2	symbiont-free	*P. aurelia*				***	NS
RanNy1602-04	*M. polyxenophila*	*P. caudatum*	+	16S	CS	***	CS
RanNy1602-11	*M. polyxenophila*	*P. caudatum*	+	16S	CS	***	CS
RanNy1602-12	*M. polyxenophila*	*P. caudatum*	+	16S	CS	***	CS
RanNy1602-18	*M. polyxenophila*	*P. caudatum*	+	16S *	SSz	COI *	SSz
RanNy1602-19	*M. polyxenophila*	*P. caudatum*	+	16S *	SSz	COI *	SSz
RanNy1602-23	*M. polyxenophila*	*P. caudatum*	+	16S *	SSz	COI *	SSz
RanNy1602-24	*M. polyxenophila*	*P. caudatum*	+	16S *	SSz	COI *	SSz
RanNy1602-30	*M. polyxenophila*	*P. caudatum*	+	16S *	SSz	COI *	SSz
RanNY1606			no paramecia detected				
RanNy1607-01	*M. polyxenophila*	*P. caudatum*		**		***	SG
RanNy1607-02	*M. polyxenophila*	*P. caudatum*		**		***	SG
RanNy1607-03	*M. polyxenophila*	*P. caudatum*		**		***	SG
RanNY1607-05	*M. polyxenophila*	*P. caudatum*	+	16S	JS	COI	JS
RanNY1607-07	*M. polyxenophila*	*P. caudatum*	+	16S	JS	COI	JS
RanNy1607-08	*M. polyxenophila*	*P. caudatum*	+	16S *	SSw	COI *	SSw
RanNY1607-09	*M. polyxenophila*	*P. caudatum*	+	16S *	JS	COI *	JS
RanNy1607-10	*M. polyxenophila*	*P. caudatum*	+	16S *	SSw	COI *	SSw
RanNY1607-11	*M. polyxenophila*	*P. caudatum*	+	16S *	JS	COI *	JS
RanNY1607-12	*M. polyxenophila*	*P. caudatum*	+	16S *	JS	COI *	JS
RanNY1607-13	*M. polyxenophila*	*P. caudatum*		**		***	AP
RanNY1607-14	*M. polyxenophila*	*P. caudatum*		**		***	AP
RanNy1607-15	*H. undulata*	*P. caudatum*	+	16S *	DH	COI *	DH
RanNy1607-16	*M. polyxenophila*	*P. caudatum*		**		***	AP
RanNy1607-17	symbiont-free	*P. caudatum*				***	AP
RanNy1607	-	*P. bursaria*	-	-	-	***	BS
RanNy1702-01	*C. varicaedens*	*P. aurelia*	+	16S	JJ	***	NH
RanNy1702-02	*C. varicaedens*	*P. aurelia*	+	16S	NH	***	NH
RanNy1702-03	*C. varicaedens*	*P. aurelia*	+	16S	NH	***	NH
RanNy1702-04	*C. varicaedens*	*P. aurelia*	+	16S	NH	***	NH
RanNy1702-05	*C. varicaedens*	*P. aurelia*	+	16S	NH	***	NH
RanNy1702-06	*C. varicaedens*	*P. aurelia*	+	16S	JJ	***	NH
RanNy1702-07	*C. varicaedens*	*P. aurelia*	+	16S	NH	***	NH
RanNy1702-L1	*C. varicaedens*	*P. biaurelia*	+	16S *	DH	COI *	DH
RanNy1702-L2	*C. varicaedens*	*P. biaurelia*	+	16S *	DH	COI *	DH
RanNy1702-L3	*C. varicaedens*	*P. biaurelia*	+	16S	DH	COI	DH
RanNy1702-L4	*C. varicaedens*	*P. biaurelia*	+	16S *	DH	COI *	DH
RanNy1702-L6	*C. varicaedens*	*P. aurelia*	+	16S	JJ	***	JJ
RanNy1702-M1	*C. varicaedens*	*P. biaurelia*	+	16S *	DH	COI *	DH
RanNy1702-M2	*C. varicaedens*	*P. biaurelia*	+	16S	DH	COI	DH

Supplementary Table S1-2: Established monoclonal *Paramecium* strains collected at different time points from Lake Nymphensee.

Strain	Symbiont	Host	Full cycle rRNA approach	Symbiont ID	by	Host ID	by
RanNy1702-M3	*C. varicaedens*	*P. biaurelia*	+	16S *	DH	COI *	DH
RanNy1702-M4	*C. varicaedens*	*P. biaurelia*	+	16S *	DH	COI *	DH
RanNy1702-M5	*C. varicaedens*	*P. aurelia*	+	16S	JJ	***	JJ
RanNy1702-M6	*C. varicaedens*	*P. aurelia*		**	AP	***	AP
RanNy1702-M7	*C. varicaedens*	*P. biaurelia*	+	16S *	SSw	COI *	SSw
RanNy1702-M8	*C. varicaedens*	*P. biaurelia*	+	16S *	SSw	COI *	SSw
RanNy1704		no paramecia detected					
RanNy1705	-	*P. bursaria*	-	-	-	***	BS
RanNy1705-01	*M. polyxenophila*	*P. caudatum*	+	16S *	JB	COI *	JB
RanNy1705-02	*M. polyxenophila*	*P. caudatum*	+	16S *	DH	COI *	DH
RanNy1705-03	*M. polyxenophila*	*P. caudatum*	+	16S *	DH	COI *	DH
RanNy1705-05	*M. polyxenophila*	*P. caudatum*	+	16S *	DH	COI *	DH
RanNy1705-06	symbiont-free	*P. caudatum*				***	DH
RanNy1705-07	symbiont-free	*P. caudatum*				***	SSw
RanNy1705-12	*M. polyxenophila*	*P. caudatum*	+	16S	JJ	***	JJ
RanNy1705-14	*M. polyxenophila*	*P. caudatum*		**		***	JJ
RanNy1705-15	*M. polyxenophila*	*P. caudatum*		**		***	AP
RanNy1709-01	symbiont-free	*P. caudatum*				***	DH
RanNy1709-2P1	symbiont-free	*P. caudatum*				***	DH
RanNy1709-2P2	symbiont-free	*P. caudatum*				***	DH
RanNy1709-2P4	symbiont-free	*P. caudatum*				***	DH
RanNy1709-2P5	symbiont-free	*P. caudatum*				***	DH
RanNy1709-2P6	symbiont-free	*P. caudatum*				***	DH
RanNy1709-2P7	symbiont-free	*P. caudatum*				***	DH
RanNy1709-2P8	symbiont-free	*P. caudatum*				***	DH
RanNy1709-2P9	symbiont-free	*P. caudatum*				***	DH
RanNy1709-2P10	symbiont-free	*P. caudatum*				***	DH
RanNy1709-2P11	symbiont-free	*P. caudatum*				***	DH
RanNy1709-2P12	symbiont-free	*P. caudatum*				***	DH
RanNy1709-03	symbiont-free	*P. caudatum*				***	DH
RanNy1709-04	symbiont-free	*P. caudatum*				***	DH
RanNy1709-05	symbiont-free	*P. caudatum*				***	DH
RanNy1709-06	symbiont-free	*P. caudatum*				***	DH
RanNy1709-07	symbiont-free	*P. caudatum*				***	DH
RanNy1709-08	symbiont-free	*P. caudatum*				***	DH
RanNy1709-09	symbiont-free	*P. caudatum*				***	DH
RanNy1709-10	symbiont-free	*P. caudatum*				***	DH
RanNy1709-11	symbiont-free	*P. caudatum*				***	DH
RanNy1709-12	symbiont-free	*P. caudatum*				***	DH
RanNy1801-01	symbiont-free	*P. caudatum*				***	DH
RanNy1801-02	symbiont-free	*P. caudatum*				***	DH
RanNy1804-01	*M. polyxenophila*	*P. caudatum*		**		COI *	FF
RanNy1804-06	symbiont-free	*P. caudatum*				COI *	FF
RanNy1804-07	symbiont-free	*P. caudatum*				COI *	FF
RanNy1804-08	symbiont-free	*P. caudatum*				COI *	FF
RanNy1804-09	symbiont-free	*P. caudatum*				COI *	FF
RanNy1804-10	symbiont-free	*P. caudatum*				***	SSw
RanNy1805-01	symbiont-free	*P. caudatum*				***	SSw
RanNy1805-02	symbiont-free	*P. caudatum*				***	SSw
RanNy1805-03	symbiont-free	*P. caudatum*				***	SSw
RanNy1805-04	symbiont-free	*P. caudatum*				COI *	SSf
RanNy1805-05	symbiont-free	*P. caudatum*				***	SSw
RanNy1805-06	symbiont-free	*P. caudatum*				***	SSw
RanNy1805-07	*M. polyxenophila*	*P. caudatum*	+	16S *	SSw	COI *	SSw
RanNy1805-08	*M. polyxenophila*	*P. caudatum*	+	16S *	SSw	COI *	SSw

Supplementary Table S1-3: Established monoclonal *Paramecium* strains collected at different time points from Lake Nymphensee.

Strain	Symbiont	Host	Full cycle rRNA approach	Symbiont ID	by	Host ID	by
RanNy1805-09	symbiont-free	*P. caudatum*				***	SSw
RanNy1805-10	symbiont-free	*P. caudatum*				***	SSw
RanNy1805-11	symbiont-free	*P. caudatum*				***	SSw
RanNy1805-12	*M. polyxenophila*	*P. caudatum*		**		***	SSw
RanNy1805-13	symbiont-free	*P. caudatum*				***	SSw
RanNy1805-14	*M. polyxenophila*	*P. caudatum*	+	16S *	SSw	COI *	SSw
RanNy1805-15	symbiont-free	*P. caudatum*				***	SSw
RanNy1805-16	symbiont-free	*P. caudatum*				COI *	SSw
RanNy1805-27	*M. polyxenophila*	*P. caudatum*				***	AP
RanNy1808		no paramecia detected					
RanNy1902-01	*M. polyxenophila*	*P. caudatum*	+	16S	SSw	COI	SSw
RanNy1902-02	*M. polyxenophila*	*P. caudatum*	+	16S	SSw	COI	SSw
RanNy1902-03	*M. polyxenophila*	*P. caudatum*	+	16S	SSw	COI	SSw
RanNy1902-04	*M. polyxenophila*	*P. caudatum*	+	16S	SSw	COI	SSw
RanNy1902-05	*M. polyxenophila*	*P. caudatum*	+	16S	SSw	COI	SSw
RanNy1902-07	*M. polyxenophila*	*P. caudatum*	+	16S *	JB	COI *	JB
RanNy1902-10	*M. polyxenophila*	*P. caudatum*		**		***	AP
RanNy1902-12	*M. polyxenophila*	*P. caudatum*	+	16S	SSf	COI	SSf
RanNy1902-13	*M. polyxenophila*	*P. caudatum*	+	16S	SSf	COI	SSf
RanNy1902-14	*M. polyxenophila*	*P. caudatum*	+	16S *	SSf	COI *	SSf
RanNy1902-19	*M. polyxenophila*	*P. caudatum*	+	16S *	JB	COI *	JB
RanNy1902-21	*M. polyxenophila*	*P. caudatum*	+	16S *	JB	COI *	JB
RanNy1902-27	*M. polyxenophila*	*P. caudatum*	+	16S *	JB	COI *	JB
RanNy1902-02		*P. bursaria*				***	AP

Supplementary Table S2: Probes used for characterization of bacterial endosymbionts via fluorescence *in situ* hybridization (FISH).

Probe	Nucleotide sequence (5' – 3')	Target	Reference
EUB338	GCT GCC TCC CGT AGG AGT	Domain *Bacteria*	Amann et al 1990
Alf1b	CGT TCG YTC TGA GCC AG	*Alphaproteobacteria*	Manz et al 1992
Rick527	CCC CTC CGT CTT ACC G	*Rickettsiaceae / Caedimonas varicaedens*	Vannini et al 2005
Megenus-487	GCC GGG GCT TTT TCT GTT GGT	all genus members "*Ca*. Megaira"	Lanzoni et al 2019
MegPol436	TTA TCT TTC CAA CTA AAA G	"*Ca*. Megaira polyxenophila"	Schrallhammer et al 2013
Ctaenio998	CTC TCT CGT CTT CTA TGG	*Caedibacter taeniospiralis*	Beier et al 2002
CC23a	TTC CAC TTT CCT CTC TCG	*Caedimonas varicaedens*	Springer et al 1993
Holosp1	TTC CAC TTT CCT CTA CCG	*Holospora*	Fokin et al 1996

Supplementary Table S3: Oligonucleotides used for molecular characterization of both *Paramecium* and endosymbiotic bacteria.

Primer	Sequence [5'-3']	Annealing temperature [°C]	Reference
COX-L	TGA TTA GAC TAG AGA TGG C	52.4	Barth et al., 2006
COX-H	GAA GTT TGT CAG TGT CTA TCC	55.9	Barth et al., 2006
16S_alphaF19b	CCT GGC TCA GAA CGA ACG	51.0	Vannini et al., 2004
16S_alphaR1517	TGA TCC AGC CGC AGG TTC	54.0	Vannini et al., 2004
16S_R1488Holo	TAC CTT GTT ACG ACT TAA CC	53.2	Boscaro et al., 2013
16S_F49alphasym	TAA CAC ATG CAA GTC GAA C	52.4	Boscaro et al., 2013
16S_alphaF114Holo	TGA GTA ACG CGT GGG AAT C	56.7	Boscaro et al., 2013
16S_F69_Meg	AAC AAA ATT GGG GCT TGC	62.4	this study
16S_R1443_Meg	CTG CCT CTT ACG TTA GC	53.3	this study

Supplementary Table S4: Oligonucleotides used for sequencing of the cytochrome C oxidase I gene of paramecia and the 16S rRNA gene for bacterial endosymbionts.

Primer	Sequence [5'-3']	Reference
COX-L	TGA TTA GAC TAG AGA TGG C	Barth et al., 2006
COX-H	GAA GTT TGT CAG TGT CTA TCC	Barth et al., 2006
16S_Bac_F343_NonDeg	TAC GGG AGG CAG CAG	Vannini et al. 2004
16S_Bac_F515_NonDeg	GTG CCA GCA GCC GCG GT	Vannini et al. 2004
16S_Bac_F785_NonDeg	GGA TTA GAT ACC CTG GTA	Vannini et al. 2004
16S_Bac_R785_NonDeg	TAC CAG GGT ATC TAA TCC	Vannini et al. 2004
16S_Bac_F1099_NonDeg	GCA ACG AGC GCA ACC C	Vannini et al. 2004

Supplementary Table S5: Primer pairs used for amplification of bacterial 16S rRNA gene.

PCR	Primer pairs	Annealing temperatures [°C]
PCR 1 (initial)	16S_alphaF19b<br>16S_alphaR1517	x=60, y=58, z=55
snPCR 1a	16S_alphaF19b<br>16S_R1488Holo	x=58, y=55, z=53
snPCR 1b	16S_F49alphasym<br>16S_alphaR1517	x=58, y=55, z=52
nPCR 1	16S_F114_HoloCae<br>16S_R1488	x=60, y=57, z=53
PCR 2	16S_alphaF114Holo<br>16S_alphaR1517	x=59, y=57, z=55
PCR 3	16S_F69_Meg<br>16S_R1443_Meg	x=54, y=53, z=52

Supplementary Table S6: PCR program used for amplification of the bacterial 16S rRNA gene. Annealing temperatures (x – z) are defined in accordance with primer combinations in Supplementary Table S4.

Steps	Temperature [°C]	Time [min]	Cycles [n]
Initial denaturation	94	3	1
Denaturation	94	0.5	
Annealing	x	0.5	5
Extension	72	1.5	
Denaturation	94	0.5	
Annealing	y	0.5	10
Extension	72	1.5	
Denaturation	94	0.5	
Annealing	z	0.5	15
Extension	72	1.5	
Final extension	72	10	1

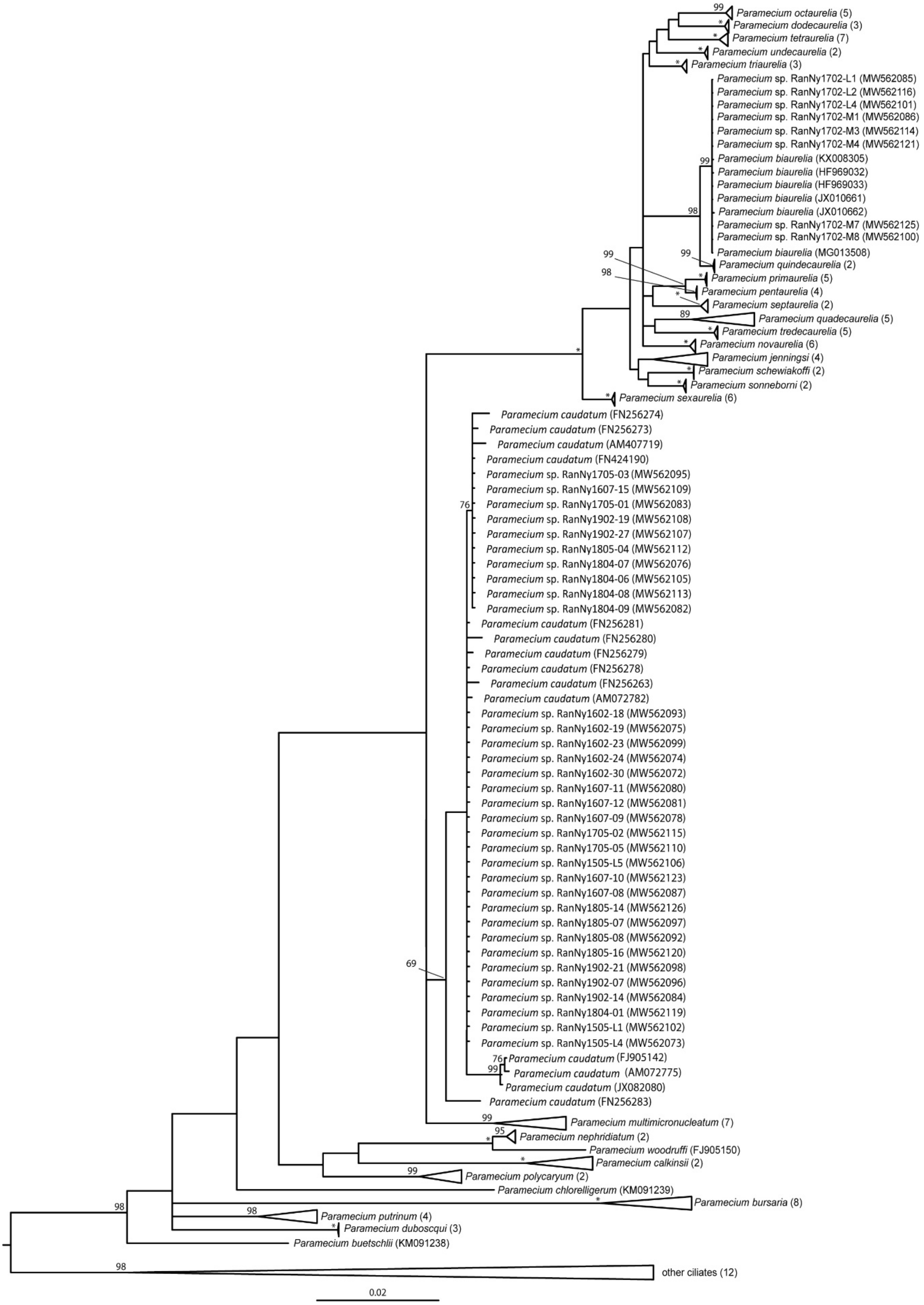

Supplementary Figure S1:

Molecular phylogeny of the genus *Paramecium* based on COI gene sequences. Maximum Likelihood tree based on 525 aligned positions calculated by IQ-Tree. TVM+F+I+G4 was used as evolutionary model. 12 sequences of other ciliate species were used as outgroup. Ultrafast boostrap support values shown if equal or higher than 50 %. Asterisks indicate maximum support. All RanNy sequences were newly characterized. Trapezoid

forms represent collapsed sequence groups, numbers in brackets indicate the number of collapsed sequences included.

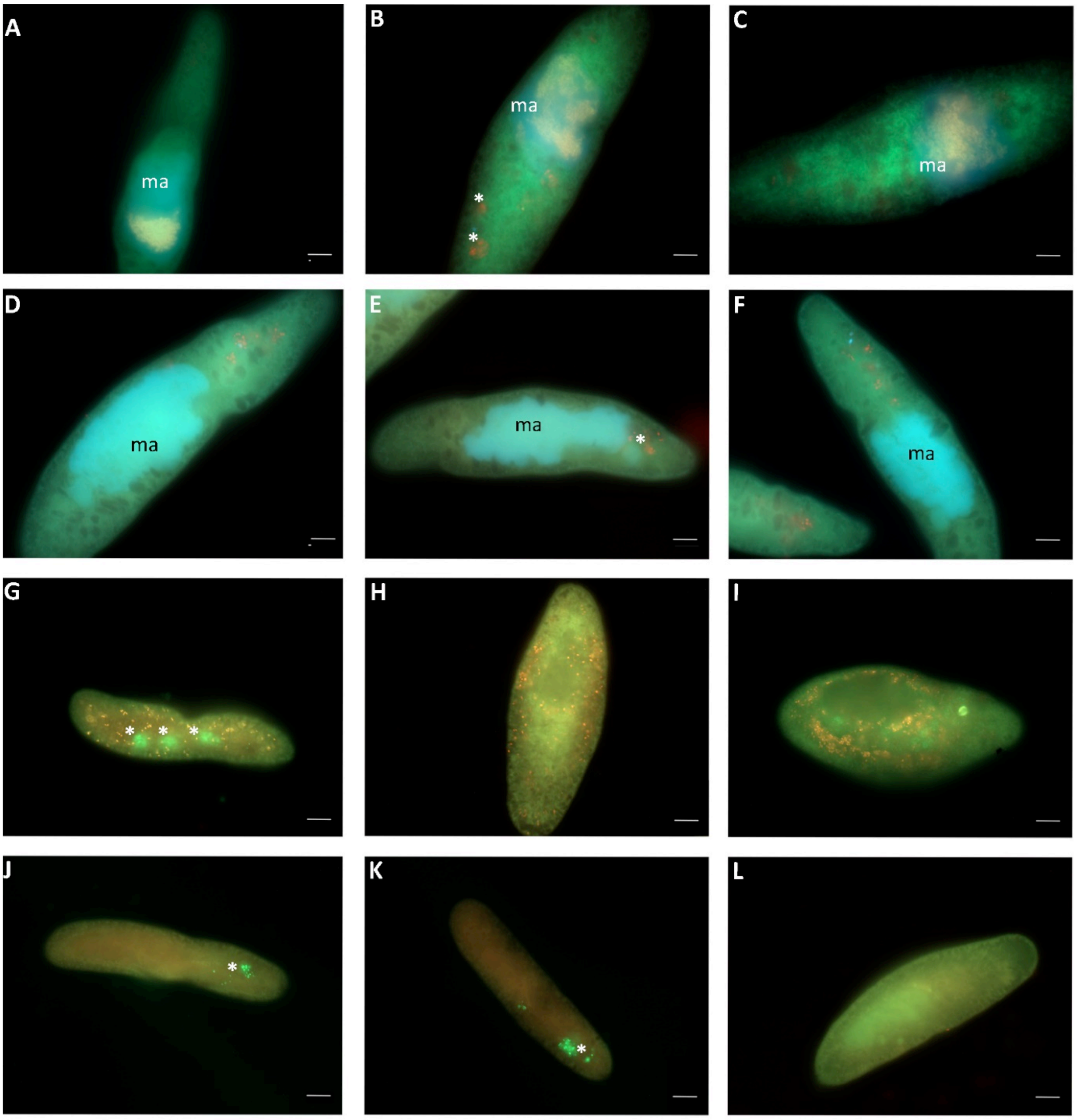

Supplementary Figure S2: Fluorescence *in situ* hybridization of *Paramecium* strains used in the competitive growth experiment to confirm their infection status. **(A - C)** *Paramecium caudatum* RanNy1505-L4 infected with "*Ca*. Megaira polyxenophila" in the macronucleus, **(D - F)** symbiont-free *P. caudatum* RanNy1804-09, **(G - I)** *Paramecium biaurelia* RanNy1702-M5 infected with *Caedimonas varicaedens* and **(J – L)** *P. biaurelia* RanNy1702-M5-AB cured from *Caedim. varicaedens*. **(A, D, G, J)** represent the infection status at the beginning of the experiment, after 96 hours **(B, E, H, K)**, and after 260 hours **(C, F, I, L)**. **(A - L)** Merge of signals from fluorescence *in situ* hybridization applying the universal probe EUB338 (green or red signal, depending on the used fluorescence dye for the universal probe), a second probe specific for species- or genus-level (**A - C** Megenus-487, green signal, MegPol436, red signal; **G – I** Cc23a, red signal), and DAPI (blue signal) **(A - F)**

visualizing the macronucleus (ma). Asterisks indicate food vacuoles. Scale bars = 10µm. "*Ca.*" stands for "*Candidatus*".

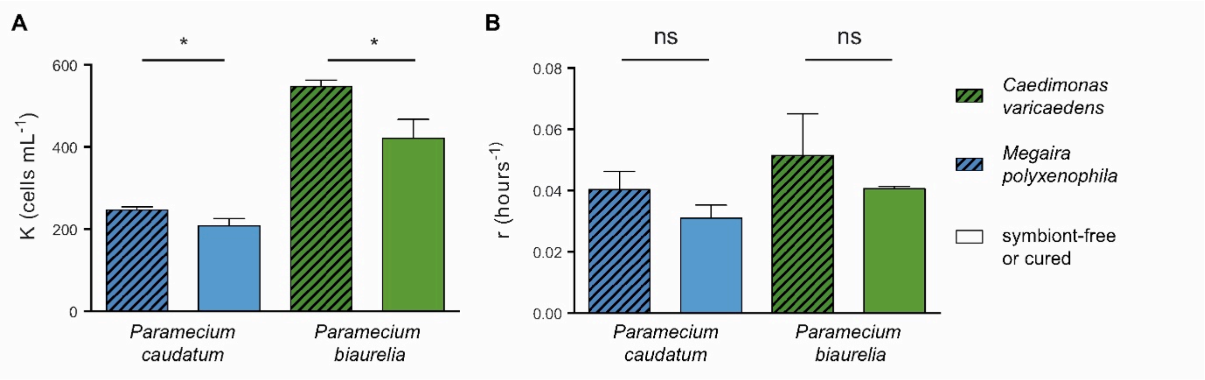

Supplementary Figure S3: Comparison of the carrying capacities K and growth rates r between the infected and symbiont-free or aposymbiotic *Paramecium* strains used in the competitive growth experiment. **(A)** Comparison of carrying capacities and **(B)** of growth rates of infected versus symbiont-free or aposymbiotic paramecia. Dashed bars indicate infected *Paramecium caudatum* with "*Ca.* Megaira polyxenophila" (blue) and *Paramecium biaurelia* infected with *Caedimonas varicaedens* (green), respectively. Plain coloured bars represent symbiont-free (*P. caudatum* RanNy1804-09, blue) and aposymbiotic *P. biaurelia* RanNy1702-M5-AB, cured from *C. varicaedens* (green). Shown is the mean cell number calculated over three replicates, error bars represent SD. Asterisks indicate $p < 0.05$. "*Ca.*" stands for "*Candidatus*".

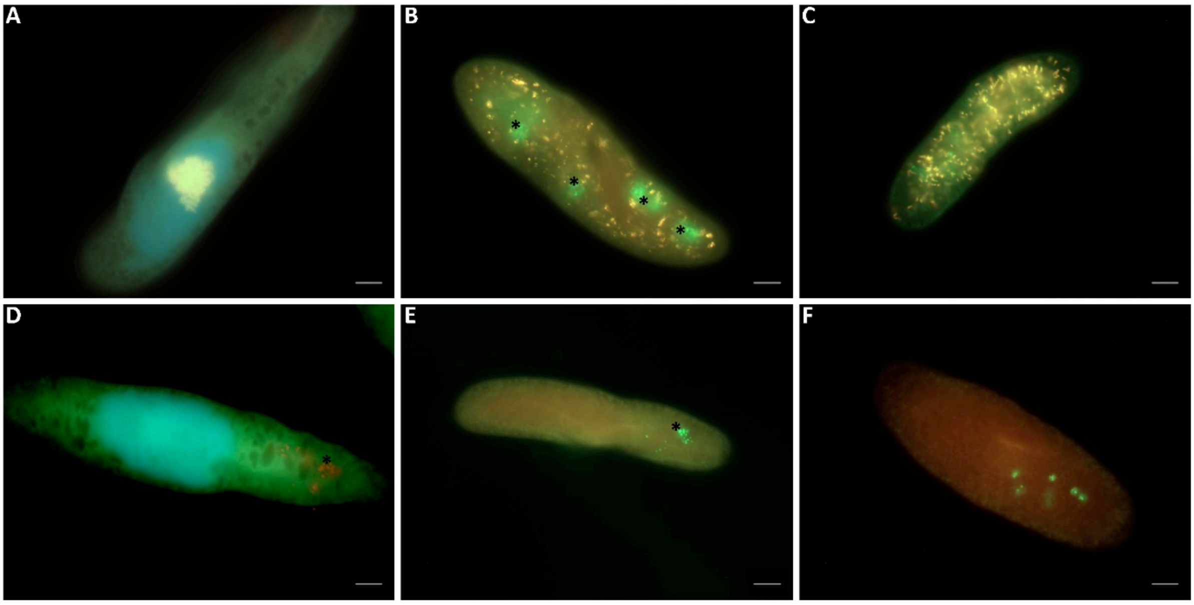

Supplementary Figure S4: Fluorescence *in situ* hybridization of infected ("killer") and symbiont-free or aposymbiotic ("sensitives") *Paramecium* strains. **(A)** *Paramecum caudatum* RanNy1505-L4 harbouring "*Ca.* Megaira polyxenophila" in the macronucleus, **(B)** *Paramecium biaurelia* RanNy1702-M5 infected with cytoplasmic *Caedimonas varicaedens* and **(C)** *Paramecium tetraurelia* 51K with *Caedibacter taeniospiralis* in the cytoplasm as well. **(D)** shows symbiont-free *P. caudatum* RanNy1804-09, **(E)** aposymbiotic *P. biaurelia* RanNy1702-M5-AB cured from *Caedim. varicaedens* and **(F)** *P. tetraurelia* 51S cured from *Caedib. taeniospiralis*. **(A - F)** Merge of signals from fluorescence *in situ* hybridization applying the universal probe EUB338, a second probe specific for species- or genus-level (**A & D** Megenus-487, green signal, MegPol436, red signal; **B & E** Cc23a, red signal; **C & F** Ctaenio998, red signal), and DAPI (blue signal) **(A, D)** visualizing

the macronucleus (ma). Asterisks indicate food vacuoles, green signal indicates *Raoultella planticola*. Scale bars = 10µm. "*Ca.*" stands for "*Candidatus*".

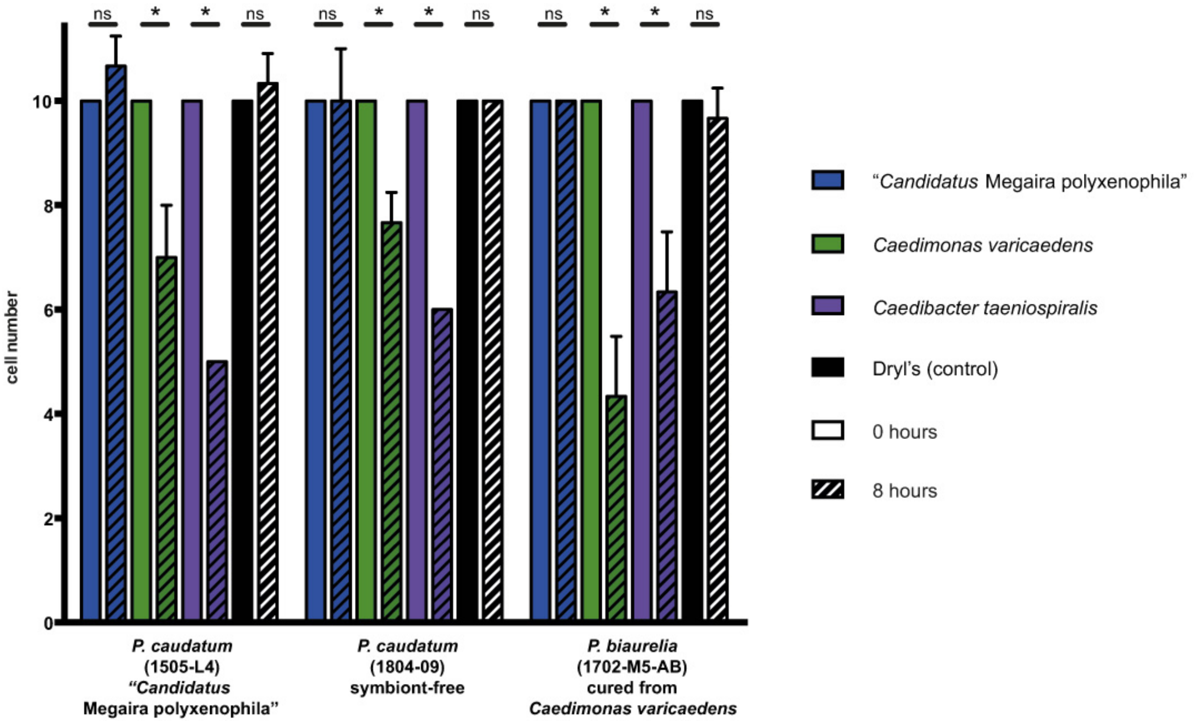

Supplementary Figure S5: Lethal potential of „*Candidatus* Megaira polyxenophila", *Caedimonas varicaedens* and *Caedibacter taeniospiralis*. Potential lethal effects of the different released bacterial endosymbionts. The number of surviving *Paramecium* cells after eight hours of exposure to bacteria (maximal observed effect, dashed bars) released after mechanical lysis of the host cell was compared to the respective initial cell numbers (solid bars). Depicted are the mean cell numbers of three replicates ± SD. For statistical analysis, a two-way ANOVA followed by Tukey's multiple comparisons test was performed with $p \leq 0.05$.
